# Chronic instability of the anterior tibiofibular syndesmosis of the ankle. Arthroscopic findings and results of anatomical reconstruction

**DOI:** 10.1186/1471-2474-12-212

**Published:** 2011-09-27

**Authors:** Marc L Wagener, Annechien Beumer, Bart A Swierstra

**Affiliations:** 1 Department of Orthopaedics, Sint Maartenskliniek, P.O. Box 9011, 6500GM Nijmegen, the Netherlands

## Abstract

**Background:**

The arthroscopic findings in patients with chronic anterior syndesmotic instability that need reconstructive surgery have never been described extensively.

**Methods:**

In 12 patients the clinical suspicion of chronic instability of the syndesmosis was confirmed during arthroscopy of the ankle. All findings during the arthroscopy were scored. Anatomical reconstruction of the anterior tibiofibular syndesmosis was performed in all patients. The AOFAS score was assessed to evaluate the result of the reconstruction. At an average of 43 months after the reconstruction all patients were seen for follow-up.

**Results:**

The syndesmosis being easily accessible for the 3 mm transverse end of probe which could be rotated around its longitudinal axis in all cases during arthroscopy of the ankle joint, confirmed the diagnosis. Cartilage damage was seen in 8 ankles, of which in 7 patients the damage was situated at the medial side of the ankle joint. The intraarticular part of anterior tibiofibular ligament was visibly damaged in 5 patients. Synovitis was seen in all but one ankle joint. After surgical reconstruction the AOFAS score improved from an average of 72 pre-operatively to 92 post-operatively.

**Conclusions:**

To confirm the clinical suspicion, the final diagnosis of chronic instability of the anterior syndesmosis can be made during arthroscopy of the ankle. Cartilage damage to the medial side of the tibiotalar joint is often seen and might be the result of syndesmotic instability. Good results are achieved by anatomic reconstruction of the anterior syndesmosis, and all patients in this study would undergo the surgery again if necessary.

## Background

The distal tibiofibular syndesmosis consists of the interosseous tibiofibular ligament (IL), the anterior tibiofibular ligament (ATiFL), and the posterior tibiofibular ligament (PTiFL) with the transverse ligament (TL) [1-3].

In 1% to 11% of the soft tissue injuries of the ankle, the syndesmosis is reported to be affected [4,5]. Injury to the syndesmosis occurs through rupture or bony avulsion of the syndesmotic ligament complex [2,6,7]. These injuries result most often from an external rotation trauma [5,8]. Other trauma mechanisms that have been found to cause syndesmotic injury are abduction, dorsiflexion and inversion [5,9,10]. During external rotation of the foot the fibula is translated posteriorly and rotated externally, which results in a high tension of the ATiFL. This may attribute to the isolated rupture of the ATiFL [11].

Rupture of the ATiFL, in its turn, causes instability of the ankle mortise [2,6,10-14]. Following an injury to the syndesmosis, pain during activity, a feeling of instability and weakness of the ankle (most often without 'frank' giving way) are also commonly experienced symptoms. Furthermore, tenderness over the ATiFL, and swelling at the level of the syndesmosis, a 'high sprain', are common signs [8,15-17]. The recovery period after a rupture of the distal tibiofibular syndesmosis is described to be considerably longer than in patients suffering from a 'normal' lateral ankle sprain [5,8,15,18].

While complete instability of the syndesmosis may be recognised during fluoroscopy or on radiographs by diastasis of the mortise, the diagnosis 'chronic instability of the anterior syndesmosis' can be difficult to make. It is an 'open book' injury of the mortise in which the fibula rotates externally allowing a greater range of motion of the talus [3]. In patients with chronic complaints after an injury, the symptoms and signs as mentioned above in combination with the mechanism of trauma, and a thorough physical examination, which includes tests to assess the integrity of the ligaments of the syndesmosis, should arouse a strong suspicion of chronic instability anterior syndesmosis [19,20]. There are specific syndesmosis stress tests. The *squeeze test*, which is performed by compressing the fibula to the tibia at the midpoint of the calf. This test is considered positive when proximal compression produces distal pain in the area of the distal tibiofibular joint [5]. In the *external rotation stress test *as described by Boytim et al. [8], external rotation stress is applied to the ankle in a neutral position with the knee flexed 90°. A positive test result is noted when pain in the area of the distal tibiofibular joint is felt. The *fibula translation test *is considered positive when pain is felt over the syndesmosis or at the deltoid ligament on translating the fibula with respect to the tibia in the anterior posterior plane [21]. In the *Cotton test *the talus is 'rocked' from side to side in the ankle mortise by applying alternating medial and lateral stress to the talus [22]. When positive, a characteristic click may be felt in the ankle mortise and the patient experiences pain [13].

When, based on medical history and physical examination syndesmotic injury is suspected, but standard radiographs of the ankle show no indication that syndesmotic injury is present or the diagnosis is still open to debate, additional evaluation of the syndesmosis can be desirable. During arthroscopy of the ankle, injury of the anterior syndesmosis can be confirmed with more certainty [23-27]. Torn parts of the anterior syndesmotic ligament can often be seen. Inserting a probe into the distal tibiofibular joint, and easy turning the transverse 3 mm end of the probe around its long axis in the syndesmosis are mentioned as ways to assess the integrity of the syndesmosis [11,18].

Reconstruction of the anterior syndesmosis to regain stability of the ankle mortise can be performed in patients with chronic instability. At the time of presentation of these patients to our hospital, literature showed no proof that chronic anterior syndesmotic injury could be adequately diagnosed on MRI, therefore this was not performed. MRI was only performed to exclude other pathology.

The aim of this study is to describe the findings during arthroscopy of the ankle in patients with chronic anterior syndesmotic instability and the clinical findings before and after anatomical reconstruction of the ATiFL when injury of the anterior syndesmosis is confirmed during arthroscopy.

## Methods

This is a prospective review of 12 patients in whom during the arthroscopy of the ankle anterior instability of the distal tibiofibular joint was confirmed [11,18,27]. There were 3 men and 9 women with a mean age of 32 years (range 17 to 54 years) at the moment of arthroscopy. In 11 of these patients the clinical suspicion of injury of the syndesmosis, based on medical history and physical examination as described by Beumer et al. [20] had been the indication for arthroscopy. In the other patient suspected osteochondritis dissecans was the indication for the arthroscopy.

The physical examination before the arthroscopy included inspection for swelling and tenderness at the level of the syndesmosis, and evaluation of ankle range of motion, the alignment of the ankle, and the specific syndesmotic stress tests as described in the introduction, except for the Cotton test which was only performed during the follow-up. Furthermore, the anterior drawer test was performed to rule out lateral instability of the ankle, and clinical evaluation to exclude abnormal ligament laxity according to Beighton et al. [28] was performed. The Clinical Rating Index for Ankle-Hindfoot [29] was scored in all patients pre- and post-operatively. A score of 95 to 100 was scored as excellent, 85 to 94 as good, 65 to 84 as fair, and less than 65 was scored as poor. Standard weight bearing anterior-posterior and lateral radiographs were made in all patients. These were evaluated for osseous abnormalities, and the presence of heterotopic ossifications. Further assessment of parameters indicating syndesmotic instability was performed. These parameters are: unilateral absence of tibiofibular overlap in the AP radiograph [30,31], and a medial clear space that is larger than the superior clear space, furthermore the distance between the medial side of the fibula and the deepest point of the tibial incisure should not exceed 5 mm [19,30]. Patients' details are displayed in Table 1.

**Table 1:** Results

Patent No	Age (y)	Gender	Side	Clinical rating index preop	Rating preop	Radiograph preop	Synovitis visible	Sydesmosis accessible for test probe	Tibiofibular ligament visibly damaged	Cartilage damage	Clinical rating index postop	Rating postop	Sefton score postop	Radiograph postop
**1**	33	M	Right	85	Good	-	+ (S)	+	0	MTa	100	Excellent	1	-
**2**	36	F	Right	72	Fair	-	+ (S)	+	+	-	90	Good	1	-
**3**	45	F	Left	59	Poor	-	+ (S&J)	+	0	MTa	87	Good	2	-
**4**	18	F	Right	75	Fair	-	+ (S&J)	+	+	-	100	Excellent	1	-
**5**	26	F	Right	72	Fair	MCS+	+ (J)	+	+	MMa	100	Excellent	1	TFCS+ & TFO
**6**	37	F	Left	72	Fair	TFCS+	+ (S&J)	+	+	LTa & LTi	76	Fair	3	-
**7**	17	F	Left	75	Fair	-	+ (S)	+	-	LTi	100	Excellent	1	-
**8**	22	F	Left	75	Fair	-	-	+	0	-	85	Good	2	-
**9**	54	F	Left	61	Poor	TFCS+	+ (S&J)	+	-	-	85	Good	2	TFCS+
**10**	27	F	Left	70	Fair	TFCS+	+ (S&J)	+	-	MTi & LTi & MMa	100	Excellent	1	TFCS+
**11**	42	M	Right	72	Fair	TFCS+	+ (S)	+	-	MTa	85	Good	2	-
**12**	26	M	Left	72	Fair	-	+ (S&J)	+	+	MTa	100	Excellent	1	-

MCS+, medial clear space larger than superior clear space; TFCS+, distance between the medial side of the fibula and the deepest point of the tibial incisure of more than 5 mm; TFO+, absence of tibiofibular overlap; S, synovitis in the syndesmosis; J, synovitis in the joint; MTa, medial side of the talus; LTa, lateral side of the talus; MTi, medial side of the tibial pilon; LTi, lateral side of the tibial pilon; MMa, medial malleolus.

**Figure 1:**
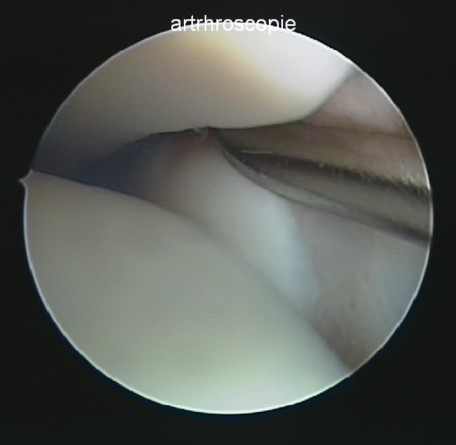
**Introduction of the 3 mm transverse end of the testprobe in the syndesmosis**.

At the time of arthroscopy, the average time after the initial injury was 24 months (range 6 to 84 months). The arthroscopies were performed in the supine position through standard anteromedial and anterolateral portals with a 30° 2.7-mm arthroscope, a tourniquet around the upper leg and an adjustable distraction. The diagnosis chronic anterior instability of the distal tibiofibular syndesmosis was made when the 3 mm transverse end of the test probe could easily be inserted and turned around in the syndesmosis [11,18,21] (Figure 1).

When present, location, and severity of damage to cartilage of the ankle joint were recorded. Articular cartilage lesions were graded according to the Outerbridge classification [32]: Grade 0, normal cartilage; Grade I, cartilage with softening and swelling; Grade II, cartilage with irregular deep fissures and villous-like cartilaginous flakes attached to the subchondral bone; Grade III, increase of the affected area with erosions down to the bare bone; Grade IV, fully exposed subchondral bone.

The presence and location of synovitis and scar tissue in the syndesmosis was recorded. When considered necessary, intra-articular shaving was performed. Ruptured portions of the intra-articular ATiFL were resected. Post-operatively full mobilisation was allowed. Arthroscopic findings were discussed with the patient and if the complaints were not resolved by resection of synovitis and torn ligament ends, advice to undergo reconstruction of the anterior syndesmosis was given.

**Figure 2:**
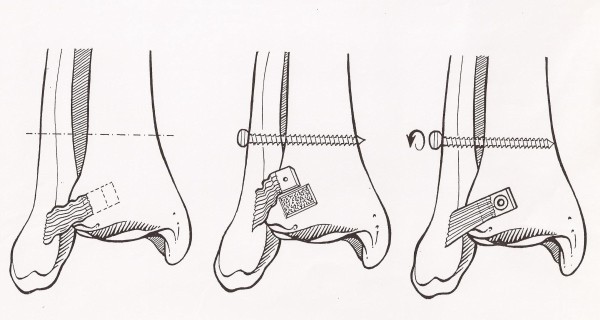
**The anatomic reconstruction of the anterior tibiofibular syndesmosis of the ankle for chronic instability (Copied with permission from Beumer A, Heijboer RP, Fontijne WPJ and Swierstra BA Late reconstruction of the anterior distal tibiofibular syndesmosis**. Good outcome in 9 patients. Acta Orthop Scand 2000; 71 (5): 519-521)

The mean time between arthroscopy and reconstructive surgery was 15 weeks (range 0 to 29 weeks). All reconstructions of the syndesmosis were performed by the same surgeon (BAS) as described by Beumer et al. [18] (Figure 2). Paying close attention to the intermediate dorsal cutaneous nerve, an anterolateral approach starting over the fibula directed towards the distal tibia was used. After identification of the slack, but continuous, anterior tibiofibular ligament the insertion in the tibia was osteotomized and mobilized with a bone block of approximately 1 × 1 cm. A gutter, running medially and slightly proximal to the original location of the bone block, was made in the tibia and after maximal compression of the mortise with a pelvic clamp the bone block was fixated in the gutter more medial and proximal than its original insertion with maximal tension on the ATiFL. Thereafter a syndesmotic screw was inserted through 4 cortices. The syndesmotic screw was removed after at least 6 weeks of non weight-bearing in a below knee cast.

All 12 patients who had had surgical reconstruction were seen for follow-up at an average of 25 (range 6 to 51) months after reconstructive surgery. The follow-up was performed by the 2 other authors who had mot performed the surgery. During follow-up the same tests as in the preoperative physical examination were performed. Standard non-weight bearing anterior-posterior and lateral radiographs were made. For comparison with the study performed by Beumer et al. [18] in which 9 patients underwent the same anatomical reconstruction of the anterior syndesmosis, a postoperative ankle score according to Sefton et al. [33] was added. The local hospital review board granted permission for this study.

## Results

All 12 patients showed an improvement of the pain and limitations, and they all would undergo the surgery again in the same circumstances.

### Clinical findings before arthroscopy

At the first visit all patients experienced pain, and limitation of the function of their ankle which subsequently resulted in limitations during their daily activities. Walking on an uneven surface caused problems in all but one patient. Demographic and clinical information on all 12 patients are given in Table 1. Information on the physical examination is given in Table 2. No patients showed signs of hyperlaxity. At initial contact the average AOFAS score was 72. Radiographs of the ankle showed only 5 ankles with an abnormality of one of the parameters that may indicate syndesmotic instability (Table 1). In 5 ankles other osseous abnormalities were seen: one old avulsion fracture of the lateral malleolus, 2 times status after bimalleolar ankle fracture, and 2 ankles showed irregularities at the level of the syndesmosis.

**Table 2:** Physical examination pre/postoperatively

Patient	Tenderness at the level of the ATiFL	Fibula translation test	External rotation stress test	Squeeze test	Anterior drawer sign	Impaired dorsal flexion
**1**	+/-	+/-	+/-	+/-	+/-	+/-
**2**	+/-	+/-	+/-	+/-	-/-	+/+
**3**	-/-	+/-	+/-	+/+	-/-	+/+
**4**	+/-	+/-	-/-	-/-	-/-	-/+
**5**	+/-	+/+	-/-	-/-	+/-	-/+
**6**	-/+	+/+	+/+	-/-	-/-	+/+
**7**	+/-	+/-	-/-	+/-	-/-	-/+
**8**	+/-	+/+	+/-	+/-	+/-	+/+
**9**	+/+	+/+	+/-	-/-	-/-	-/+
**10**	+/-	+/-	+/-	-/-	-/-	-/+
**11**	+/+	+/-	+/-	+/-	-/-	+/+
**12**	+/-	+/-	+/-	-/-	+/-	-/-

+ positive test; - negative test.

### Arthroscopic findings

In all patients included in this study the diagnosis 'chronic instability of the anterior syndesmosis' was confirmed during arthroscopy. In only 5 ankles the intra-articular part of the ATiFL was visibly damaged and 8 ankles had synovitis and/or scar tissue bulging from the syndesmosis. Cartilage damage was found in 8 ankles, all Outerbridge stage 1 except in patient 5 where the inside of the medial malleolus was bare after a fracture in the past. No treatment for the cartilage damage like forage was performed in any patient. No correlation between the time that the syndesmotic injury had occured and the presence of the cartilage damage was found. Further information on the localisation of the damaged cartilage seen during arthroscopy is given in the results summary table (Table 1).

### Follow-up after reconstruction

At an average of 25 months after reconstruction 11 patients showed an excellent or good result. In 6 patients all complaints had disappeared, all other patients showed an improvement of complaints. Only 4 patients showed slight limitations when walking on uneven ground. After the reconstruction the average AOFAS score was 92. Further information on the physical examination during follow up can be found in the physical examination table (Table 2). Standard AP and lateral radiographs after reconstruction showed 3 ankles with an abnormality of one of the parameters that may indicate syndesmotic instability (See Table 1). All patients were satisfied with the improvement of the symptoms as a result of the surgery. All patients would choose to have the reconstructive surgery again.

### Complications

There we no complications after the arthroscopies and the reconstruction surgery. Unfortunately, there was one wound infection after removal of the syndesmotic screw 6 weeks after the reconstruction. It was treated with antibiotics, proper wound care was applied, and with time the wound healed without further problems.

## Discussion

Previous studies have shown that arthroscopic evaluation of the stability of the distal tibiofibular joint is of considerable value in the diagnosis of injuries of the syndesmosis [18,21,23,24,26,27].

In the intact situation the intermalleolar distance increases with 1, 0 - 1, 1 mm during the movement from plantar flexion to dorsiflexion when the ankle is forced in dorsal flexion [34-37]. In previous studies [23,24,26] acute injury of the syndesmosis was diagnosed when a widening of 2 mm between the tibia and fibula was found during arthroscopy. Based on the knowledge that the radiographic boundaries of the syndesmosis (medial site fibula - deepest point tibial incisures) do not exceed 5 mm in non-injured specimens [30] and on the study of Bartonicek [1], in which an 2 mm wide V-shaped synovial plica is described that starts at the fibular notch and becomes narrower as it reaches the IL, in this study injury of the syndesmosis was confirmed only when the 3 mm transverse end of the probe could easily be turned around in the syndesmosis.

During arthroscopy the presence and extent of chondral pathology can easily be assessed. In this study cartilage damage in the ankle joint was seen in 10 ankles. In 1 of these ankles the damage appeared to be the direct result of an old bimalleolar ankle fracture. The cartilage damage in the other 9 ankles could be the indirect result of the instability of the ankle mortise, caused by the injury of the syndesmosis [11,12,21].

It is of interest that in the 6 patients with a positive squeeze test, during the arthroscopy no scar tissue or synovitis was found inside the syndesmosis in five of them, and only very little in one. In all patients with a negative squeeze test a considerable amount of synovitis and/or scar tissue was seen bulging out from the syndesmosis. The negative result of the squeeze test could possibly be explained by an impaired mediolateral movement of the fibula during the squeeze as a result of the scar tissue filling the syndesmosis or by a buffer function of the fibrous tissue, which results in a diminished stress and thus pain.

In this study none of the specific syndesmotic stress tests was uniformly positive in the presence of a syndesmotic rupture. This is in accordance with earlier findings [20] and confirms that no definite diagnosis should be made based on the medical history and the physical examination.

Beumer et al. [30] showed that no single optimal radiographic parameter exists to assess syndesmotic integrity. In the present study the measurements performed in the standard AP and lateral radiographs of the ankle before the reconstruction showed only 5 ankles with abnormalities that could indicate injury of the syndesmosis. This shows that the diagnosis cannot be dismissed based on the absence of radiologic abnormalities. CT, ultrasound and MRI have been shown to be useful in acute syndesmotic injuries, but we are not aware of studies proving their usefulness in chronic instability.

Cartilage injury is frequently found but none of the triad of findings described by Ogilvie-Harris et al. [21], torn IL, torn PTiFL, and an avulsion of posterior tibial dome was seen is this group of patients, nor in the other 2 groups described by us [11,18], so that we must conclude that Ogilvie-Harris et al. [21] have described a different condition than 'chronic instability of the anterior syndesmosis'. This might explain why their patients recovered from symptoms without stabilisation of the mortise.

A substantially better result of the anatomical reconstruction is seen in the present study when the postoperative Sefton-score [33] is compared to the results of the study of Beumer et al. [18] in which the same surgical reconstruction was performed in nine patients. This last study also reported 3 complications in performing the reconstruction. Transient sympathic reflex dystrophy was seen in 2 patients and entrapment of the intermediate dorsal cutaneous nerve in scar tissue was seen in 1 patient. In this study we only had one complication, namely a wound infection after removal of the syndesmotic screw 6 weeks after the reconstruction. Less complications and a better result could be explained by the surgeon paying closer attention to the intermediate dorsal cutaneous nerve as advised [18], and the learning curve which is present for all surgical procedures.

The main limitations of this study concern the small number of patients, and the absence of a control group. However, syndesmotic instability was until recently an underdiagnosed and poorly defined condition, and the present study helps to clarify diagnostic and therapeutic aspects. The long duration of complaints after the initial injury makes a self-limiting natural history less obvious.

## Conclusion

The combination of the patients' medical history, physical examination, and diagnostic tests can give a good indication of the function of the syndesmotic ligaments. When syndesmotic injury is suspected based on medical history and physical examination, the diagnosis can be confirmed during arthoscopy of the ankle. This is done by inserting a probe with a 3 mm transverse end into the syndesmosis to test the width of the distal tibiofibular joint by turning the probe around its longitudinal axis. Reconstruction of the ATiFL by a tensioning procedure can give very good results even if the interosseous ligament would have been ruptured as well.

## Competing interests

The authors declare that they have no competing interests.

## Authors' contributions

MLW performed the follow-up and wrote the manuscript. AB supervised the follow-up and edited the manuscript. BAS performed all operations, initiated the study and edited the final manuscript. All authors read and approved the final manuscript

## Pre-publication history

The pre-publication history for this paper can be accessed here:

http://www.biomedcentral.com/1471-2474/12/212/prepub
